# Modulation of β-amyloid by a single dose of GSK933776 in patients with mild Alzheimer’s disease: a phase I study

**DOI:** 10.1186/alzrt249

**Published:** 2014-04-09

**Authors:** Thomas Leyhe, Niels Andreasen, Monica Simeoni, Arno Reich, Christine AF von Arnim, Xin Tong, Astrid Yeo, Shahid Khan, Amy Loercher, Michelle Chalker, Charles Hottenstein, Henrik Zetterberg, Jan Hilpert, Prafull Mistry

**Affiliations:** 1 Department of Psychiatry and Psychotherapy, University Hospital of Tübingen, Calwerstrasse 14, D-72076 Tübingen, Germany; 2 Center of Old Age Psychiatry, Psychiatric University Hospital, Wilhelm Klein-Strasse 27, CH-4012 Basel, Switzerland; 3 Karolinska Institute, Alzheimer Research Centre, Solnavägen 1, 171 77 Solna, Stockholm Sweden; 4 Quantitative Sciences, GlaxoSmithKline, Stockley Park West, 1-3 Iron Bridge Road, Uxbridge, Middlesex UB11 1BT, UK; 5 Department of Neurology, University Hospital RWTH Aachen, Pauwelstrasse 30, Aachen, Germany; 6 Department of Neurology, Ulm University, Oberer Eselsberg 45, 89081 Ulm, Germany; 7 Clinical Pharmacology Sciences & Study Operations, GlaxoSmithKline, Gunnels Wood Road, Stevenage, Hertfordshire SG1 2NY, UK; 8 Genetics, GlaxoSmithKline, Gunnels Wood Road, Stevenage, Hertfordshire SG1 2NY, UK; 9 Clinical Immunology, GlaxoSmithKline, Upper Merion, 709 Swedeland Road, King of Prussia, PA 19406, USA; 10 Global Clinical Safety and Pharmacovigilance, GlaxoSmithKline, Stockley Park West, 1-3 Iron Bridge Road, Uxbridge, Middlesex UB11 1BT, UK; 11 Drug Metabolism and Pharmacokinetics, GlaxoSmithKline, Upper Merion, 709 Swedeland Road, King of Prussia, PA 19406, USA; 12 Department of Psychiatry and Neurochemistry, Clinical Neurochemistry Laboratory, Institute of Neuroscience and Physiology, The Sahlgrenska Academy at University of Gothenburg, V-husek Mölndal, SE-431 80, Sweden; 13 UCL Institute of Neurology, Queen Square, London WC1N 3BG, UK; 14 Neuroscienceina; 15 Quante, Hertfordshire SG1 2NY, UK

## Abstract

**Introduction:**

In this study, we evaluated the safety and pharmacodynamic effects of the Fc-inactivated anti-β-amyloid (anti-Aβ) monoclonal antibody GSK933776 in patients with mild Alzheimer’s disease and mild cognitive impairment. Aβ and tau levels were investigated in cerebrospinal fluid (CSF), and the relationship between Aβ levels and Aβ modulation in plasma was explored. The feasibility of a continuous sampling method using a lumbar catheter was assessed.

**Methods:**

This trial was a phase I, open-label, uncontrolled, single-dose, exploratory experimental medicine study of intravenous GSK933776 at doses of 1 mg/kg, 3 mg/kg or 6 mg/kg (*n* = 6/group). The time course of plasma and CSF concentrations of GSK933776 and Aβ was assessed. Sample size was based on feasibility, and no formal statistical analyses were performed.

**Results:**

Following administration of GSK933776 at doses of 1 mg/kg, 3 mg/kg and 6 mg/kg, there were decreases from baseline in CSF Aβ_1–42_ (from 0 to 12 hours) by 22.8 pg/ml (6.2%), 43.5 pg/ml (9.2%) and 60.5 pg/ml (12.5%), respectively. Plasma concentrations of total Aβ_18–35_ and Aβ42_28–42_ increased immediately after infusion and CSF tau concentration increased slightly, but did not significantly change, following administration of all doses of GSK933776. Pharmacokinetics confirmed the presence of GSK933776 in the CSF, which exhibited a dose–response relationship. One patient underwent minor surgery without sequelae following a ruptured lumbar catheter.

**Conclusion:**

GSK933776 demonstrated pharmacological activity and target engagement in CSF and plasma, and the continuous sampling method via a catheter successfully assessed the Aβ changes following single-dose administration of GSK933776.

**Trial registration:**

ClinicalTrials.gov Identifier: NCT01424436. Registered 4 August 2011

## Introduction

Aggregated β-amyloid peptide (Aβ) is the hallmark of Alzheimer’s disease (AD) brain pathology. As Aβ plaques are one of the underlying pathological features of AD, significant research efforts have been directed at developing antiamyloid therapies to clear or neutralise the amyloid peptide’s toxic forms [1,2]. Immunotherapeutic approaches for the treatment of AD include active immunisation, whereby an Aβ peptide, or a fragment of it, is administered, which produces an immune response against Aβ. Activation of both cellular and humoral immunity follows, which clears amyloid deposits from the brain. Passive immunisation involves administration of exogenous, specific monoclonal antibodies against Aβ which may treat AD by acting centrally on amyloid plaques in the brain or peripherally through an Aβ “peripheral sink” mechanism. By the peripheral sink mechanism, circulating antibodies sequester Aβ and favour efflux of Aβ from the central nervous system (CNS) over influx to the CNS [3].

Immunotherapy using monoclonal antibodies directed against Aβ is considered to be the preferred method in the search for mechanistic disease-modifying treatments for AD. The key element for an immunotherapy during early clinical development is to demonstrate that the agent engages its target. In the case of anti-Aβ antibodies, the preferred target is the CNS. During the development of the human anti-Aβ antibody gantenerumab, it was initially demonstrated in mice that the antibody bound specifically to Aβ plaques [4]. In an ascending-dose, positron emission tomographic study of patients with AD and in *ex vivo* studies of human brain slices taken from patients who had AD, gantenerumab treatment resulted in reduction of brain Aβ levels, indicating that antibody–Aβ complexes had formed [4]. Cerebrospinal fluid (CSF) examinations conducted in patients with AD following administration of solanezumab, another human anti-Aβ antibody, showed a dose-dependent increase in unbound CSF Aβ_1–42_, suggesting that this antibody shifted the Aβ equilibrium sufficiently to mobilise Aβ_1–42_ from amyloid plaques [5].

Two approaches are being used in AD clinical trials to obtain CSF samples to assess drug penetration and target engagement in the CNS. The lumbar puncture technique relies on sampling at two different time points and was used to determine CSF Aβ levels and drug concentrations in a solanezumab phase II study [5]. Alternatively, CSF samples can be taken continuously by using a catheter, as demonstrated in a previous study, to measure Aβ production and clearance in the CNS directly [6,7].

GSK933776 is a humanised mouse anti-human Aβ IgG1 monoclonal antibody that binds with high affinity to the Aβ N terminus and has the Fc region engineered to substantially reduce the fixation of complement and Fc receptor binding, which is responsible for humoral adaptive immunity and cell-mediated tissue damage.

Herein we present the results of an exploratory experimental medicine study conducted to assess the effects of a single-dose exposure of GSK933776 on Aβ levels in CSF, as well as its relationship with Aβ modulation, in the plasma of patients with mild AD and mild cognitive impairment (MCI). We assessed the time course and dose-dependency of Aβ and tau changes in the first 12 hours after administration of GSK933776 as well as the feasibility of using lumbar catheters to assess these changes. The choice of dose ranges tested in this study was driven by pharmacodynamics detected in a previous study conducted in patients with AD. GSK933776 administered in three repeat doses every 4 weeks at ≥0.1 mg/kg lowered free Aβ concentrations in plasma. For total Aβ_1–42_, the final peak/trough ratio was ≤2 at GSK933776 ≥ 3 mg/kg. For total Aβ_18–35_, the final peak/trough ratio was ≤2 at 6 mg/kg (manuscript in submission). The results of our present study will support the dose selection for subsequent larger-scale clinical studies assessing the effect of GSK933776 on cognitive, global and functional endpoints.

## Methods

### Study protocol

The study protocol was reviewed and approved by the following ethics committees: Medizinische Ethik-Kommission II, der Medizinischen Fakultät Mannheim, Mannheim, Germany; Ethik-Kommission der Medizinischen Fakultät und am Universitätsklinikum Tübingen, Germany; and Regionala Etiksprovningsnamnden Stockholm, Stockholm, Sweden. The study was conducted in accordance with Good Clinical Practice and the 2008 Declaration of Helsinki. All patients provided written, informed consent to participate in the study and, separately, to undergo genetic analysis.

### Study population

Men and nonfertile women ages 50 to 85 years with a clinical diagnosis of probable mild AD, Mini Mental State Examination (MMSE) score of 20 to 26 [8] or MCI were included. All patients demonstrated a decrease in Aβ_1–42_ to <550 pg/ml in CSF and increases in total T-tau to >400 pg/ml or phosphorylated tau 181 to >70 pg/ml in CSF, which are indicative of prodromal or mild AD [9,10]. The term *prodromal Alzheimer’s disease* is used to describe patients who have a hippocampal type of amnestic disorder and a biomarker indicative of the presence of AD, but who are not functionally impaired and do not meet the criteria for Alzheimer’s dementia [11,12]. The definition of prodromal AD is similar to that of MCI based on the classification criteria proposed by the National Institute on Aging and Alzheimer’s Association work group [13].

The inclusion criteria for entry into this trial were that patients had to be in good health, determined on the basis of routine clinical laboratory test screening results, physical examinations, vital signs and 12-lead electrocardiograms (ECGs). Exclusion criteria included a history or evidence of any other CNS disorder that could be interpreted as a cause of dementia; Hachinski Ischemic Scale score >4; a magnetic resonance imaging (MRI) scan not consistent with AD or evidence of any other CNS condition; minimal vascular changes or more than three microhaemorrhagic lesions; and a history of psychiatric illness, cerebral haemorrhage, seizures or strokes during the 3 years preceding the study; and cardiovascular disease or diabetes.

### Study design

Recruitment of patients into this open-label, single-dose study (protocol number BA1113043, Clinicaltrials.gov Identifier NCT01424436) was carried out from May 2010 to December 2011 at three sites in Germany and two in Sweden. Patients were screened on day −30 prior to dosing and randomised to receive doses of GSK933776. The patients were assigned to receive doses of GSK933776 in accordance with the randomisation schedule produced using RandAll, a web server–based clinical trials system. The doses of GSK933776 were 1 mg/kg, 3 mg/kg or 6 mg/kg (*n* = 6 per group) administered by intravenous pump infusion for 1 hour. Patients were followed for up to 56 days postdosing.

The primary endpoint was measurement of the levels and temporal changes of Aβ_1–42_ in CSF after administration of GSK933776 in patients with mild AD and MCI. Secondary endpoints were measurement of the levels and temporal changes following GSK933776 administration of the following: total Aβ42_28–42_ and Aβ_18–35_ in plasma and tau and phosphorylated tau 181 in CSF, the pharmacokinetics for GSK933776 in CSF and plasma, the pharmacokinetic–pharmacodynamic (PK–PD) relationship of GSK933776 in CSF and plasma and the safety and tolerability of GSK933776.

### Pharmacodynamics sample collection and analysis

For the first four patients who received the lowest dose (1 mg/kg), blood and CSF samples were collected at hourly intervals over a 30-hour time period from approximately 11 hours predosing to 19 hours postdosing. The primary sampling time period for evaluation of baseline levels was 6 to 8 hours predosing, whereby 0 to 5 hours postdosing was considered a run-in period during which we obtained stable values. The time period from 0 to 12 hours postdosing was the focus for the postdosing assessment. This duration was initially based on plasma pharmacodynamics observed in our previous study, that is, the time of onset of lowering of free Aβ (manuscript in submission). After the first four patients completed the protocol, their Aβ levels were reviewed and the sample collection times for CSF and blood for both baseline and postdosing were adjusted as follows. CSF samples were collected hourly (3 ml per sample) from 9 hours predosing to 12 hours postdosing. Blood samples were collected hourly from 6 hours predosing to 12 hours postdosing and at follow-up: once on day 7 and once on day 56 postdosing. Total tau and phosphorylated tau 181 were measured in CSF at 5 hours predosing, at the time of dosing and at 5 hours and 12 hours postdosing. Because of the short-term exposure to GSK933776, no changes in total tau or phosphorylated tau 181 were expected.

Insertion of the lumbar catheter was done according to local hospital protocols. Continuous CSF collection was performed according to the standard operating procedures of the study and carried out by medical staff trained in this procedure. CSF Aβ_1–42_, total tau and phosphorylated tau 181 were measured using INNOTEST kits (Innogenetics/Fujirebio Europe, Ghent, Belgium). For the INNOTEST assay for Aβ_1–42_, we used 21F12 as a coating monoclonal antibody, which is specific for the C-terminal epitope 42, and 3D5 as a detection antibody, which is specific to N-terminal epitope 1. The INNOTEST assay for phosphorylated-tau 181 uses HT7 as a coating antibody, which is specific for amino acids 159 to 163, and AT270 as a detection antibody, which is specific to phosphorylated Thr181. All CSF analyses were performed batchwise by board-certified laboratory technicians who were blinded to clinical and treatment data. Intra-assay coefficients of variation were below 10%.

For plasma preparation, blood samples were processed within 30 minutes by centrifugation at 2,000 × *g* for 15 minutes at 4°C to obtain plasma. A protease inhibitor (0.02 ml; Roche Applied Science, Burgess Hill, UK) was added to 0.5 ml of plasma. Samples were then frozen upright on dry ice or in a freezer at or below −70°C. The specificities of the capture and detection antibodies used in the plasma total Aβ measurements (numbers in brackets indicate the minimal amino acid sequence detected by the plasma Aβ assay) were as follows. The combinations of the capture antibody 6F6 (amino acids 28 to 35, generated by GlaxoSmithKline (GSK)) and detection antibody 5G5 (amino acids 35 to 42; generated by GSK) detected all Aβ fragments containing an intact C terminus, such as Aβ_1–42_ and Aβ_3–42_, which is considered indicative of total Aβ42_28–42_. 6F6 and the detection antibody 4G8 (amino acids 18 to 22) (SIG-39220; Covance, Emeryville, CA, USA) detected Aβ fragments containing the amino acid sequence 18 to 35, including Aβ_1–38_, Aβ_1–40_ and Aβ_1–42_, which are considered indicative of total Aβ_18–35_. Total assays detected both free and GSK933776-bound Aβ fragments. There was no competition between the capture antibody 6FS and GSK933776, because 6F6 recognises Aβ amino acids 28 to 35 and GSK933776 recognises Aβ amino acids 1 to 5. Total levels of Aβ_18–35_ and total Aβ42_28–42_ were measured in plasma only.

### Pharmacokinetics sample collection and analysis

Blood samples were collected once prebaseline, once during GSK933776 administration, hourly for 4 hours postdosing and then 6, 8, 10 and 12 hours postdosing as well as at follow-up 7 and 56 days postdosing. CSF samples were collected every hour over a total period of 22 hours, beginning 9 hours predosing and ending 12 hours postdosing. Approximately 0.5 ml of CSF and approximately 3 ml of whole blood were collected at every time point.

Concentrations of GSK933776 were determined in plasma and CSF samples using an immunoassay that detects free GSK933776 antibody. Briefly, the humanized monoclonal antibody drug specific to GSK933776 is captured with a Aβ fragment immobilized on a microtitre plate. The plate was washed removing all nonspecific material and GSK933776 is detected using an anti-human IgG (Fc specific) monoclonal antibody conjugated to a reporter tag which is developed and detected by immunoelectrochemiluminescence, resulting in a quantifiable response.

Pharmacokinetics analyses of plasma and CSF GSK933776 were calculated by standard noncompartmental analysis using WinNonlin Pro 5.2 software (Pharsight, Mountain View, CA, USA) and were based on actual sampling times. The following parameters were measured: the first occurrence of the maximum observed plasma and CSF concentrations determined directly from raw concentration–time data (*C*_max_); the time at which *C*_max_ was observed, which was determined directly from raw concentration–time data (*t*_max_); the area under the plasma and CSF concentration–time curve to the last quantifiable concentration (AUC_0–*t*_); the area under the plasma and CSF concentration–time curve from time 0 to 12 hours postdosing (AUC_0–12_); the time of the last observed plasma concentration (*t*_last_); and the concentration at *t*_last_ (*C*_t_). The following additional parameters were included for analysis of CSF pharmacokinetics: the areas under the CSF concentration–time curve from time 0 to 4 hours postdosing AUC_0–4_ and from 5 to 12 hours postdosing AUC_5–12_ and the time prior to the first measurable (that is, nonzero) concentration in CSF (*t*_lag_).

### Safety assessments

Clinical chemistry and haematology tests, vital signs, 12-lead ECG readings and brain MRI were performed at screening, at intervals throughout the study and at follow-up examinations. Adverse events were coded using the Medical Dictionary for Regulatory Activities (http://www.meddra.org/) criteria.

### Statistical analysis

As this was an exploratory experimental medicine study, we did not perform any formal calculations of power or sample size. Sample sizes were chosen based on feasibility to allow preliminary evaluations of the safety and effects GSK933776 on Aβ and tau levels in CSF. No formal statistical analyses were performed.

#### Pharmacodynamics analyses

CSF Aβ parameters were summarised using standard descriptive statistics for a range of summary measures (absolute, absolute change from baseline, absolute percent change from baseline, weighted mean, weighted mean change from baseline and weighted mean percent change from baseline) and are presented in individual participant plots. The weighted means were derived by calculating the AUCs using the trapezoidal rule and then dividing by the actual relevant time interval. CSF parameters (tau and phosphorylated tau 181) and plasma parameters (Aβ_18–35_ and Aβ42_28–42_) were summarised (absolute) and are presented in individual participant plots Additional file 1 and Additional file 2, respectively.

#### Pharmacokinetics analyses

Concentrations of GSK933776 in plasma and CSF were summarised using standard summary statistics. Noncompartmental plasma pharmacokinetics parameters were summarised using standard descriptive statistics, which also included coefficients of variation, geometric means and 95% confidence intervals, for the geometric means.

## Results

### Patient disposition and baseline characteristics

**Figure 1:**
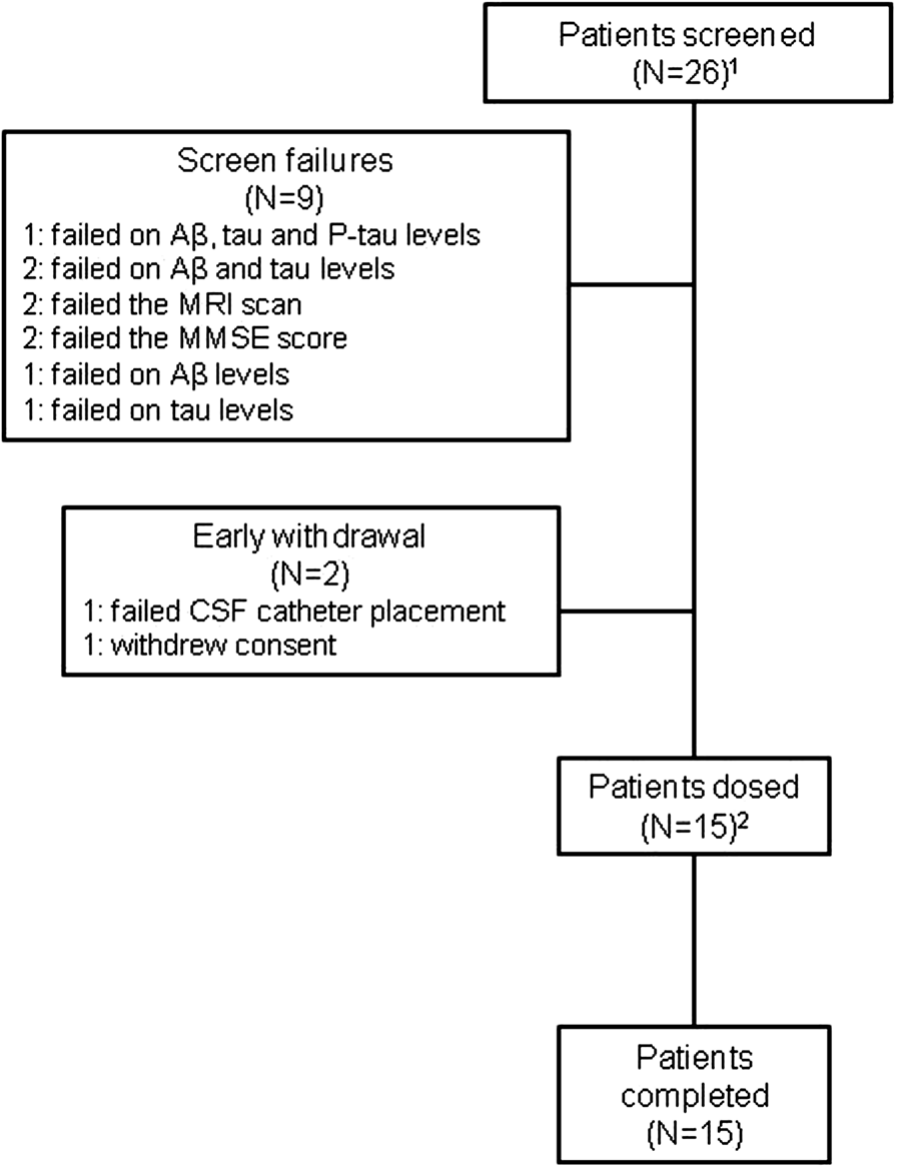
**Summary of patient screening.** Aβ: β-amyloid; CSF: Cerebrospinal fluid; MMSE: Mini Mental State Examination; MRI: Magnetic resonance imaging; P-tau: Phosphorylated tau. ^1^Three patients were screened twice therefore 29 screening evaluations were conducted. ^2^Three patients received GSK933776 at two dose levels; therefore, GSK933776 was administered 18 times.

Twenty-six patients were screened. Nine patients were excluded because they failed to meet the inclusion criteria, and two withdrew before dosing began (Figure 1). Fifteen patients received GSK933776. Three of the fifteen patients received GSK933776 at two dose levels. One patient received 1 mg/kg and 3 mg/kg one received 1 mg/kg and 6 mg/kg and one received 3 mg/kg and 6 mg/kg. For patients who were assigned to receive two dose levels, the time interval between the doses was approximately 5 months. Their baseline data are presented in Table 1. For the entire patient population, the mean age was 67.8 years (range: 57 to 70 years) and the mean body mass index was 24.18 kg/m^2^ (range: 19.6 to 27.7). Nine patients were female and six were male, and all were Caucasians. Most patients carried the *APOE* ϵ4 allele (14 (93%) of 15 patients), and six of these patients carried two copies of the allele.

**Table 1:** **Summary of patient baseline characteristics**^**a**^

		**GSK933776 dose**		
**Demographic characteristics**	**Sex**	**1 mg/kg (*n*** **= 6)**	**3 mg/kg (*n*** **= 6)**	**6 mg/kg (*n*** **= 6)**
Mean age (yr) (range)		69.0 (61 to 79)	68.3 (57 to 79)	66.0 (58 to 77)
Sex, *n* (%)	Female	2 (33)	5 (83)	3 (50)
	Male	4 (67)	1 (17)	3 (50)
Race, *n* (%)	Caucasian	6 (100)	6 (100)	6 (100)
Mean BMI kg/m^2^ (range)		23.7 (20 to 26)	23.5 (21 to 28)	25.4 (22 to 27)
*APOE* ϵ4 status, *n* (%)	E2E3	0 (0)	0 (0)	0 (0)
	E3E3	1 (17)	0 (0)	0 (0)
	E3E4	3 (50)	2 (40)/1^b^	3 (75)^c^
	E4E4	2 (33)	3 (60)	1 (25)

^a^BMI: Body mass index. ^b^One participant who entered the study and provided pharmacogenetics (PGx) consent and a sample in the 1 mg/kg dosing arm reentered the study in the 3 mg/kg dosing arm. The PGx consent and sample were obtained again unnecessarily for the 3 mg/kg dosing. The second PGx sample was subsequently destroyed to maintain consistency within the study by collecting and using only one sample per individual. ^c^Two participants who had entered the study and provided PGx consent and a sample in the 3 mg/kg dosing arm reentered the study in the 6 mg/kg dosing arm. The PGx consent and sample were not obtained again for these patients.

Mean ± standard deviation (SD) CSF concentrations for phosphorylated tau 181 were 90.6 ± 39.36 pg/ml for the GSK933776 1 mg/kg group (*n* = 4), 71.0 ± 39.75 pg/ml for 3 mg/kg (*n* = 5) and 153.9 ± 37.37 pg/ml for 6 mg/kg (*n* = 6). Mean (±SD) CSF concentrations for tau were 828.3 ± 430.29 pg/ml for the GSK933776 1 mg/kg group (*n* = 4), 668.2 ± 262.35 pg/ml for 3 mg/kg (*n* = 5) and 1,209.3 ± 262.35 pg/ml for 6 mg/kg (*n* = 6). Baseline data for patient CSF Aβ_1–42_ levels are presented in Table 2.

### Pharmacodynamics

**Figure 2:**
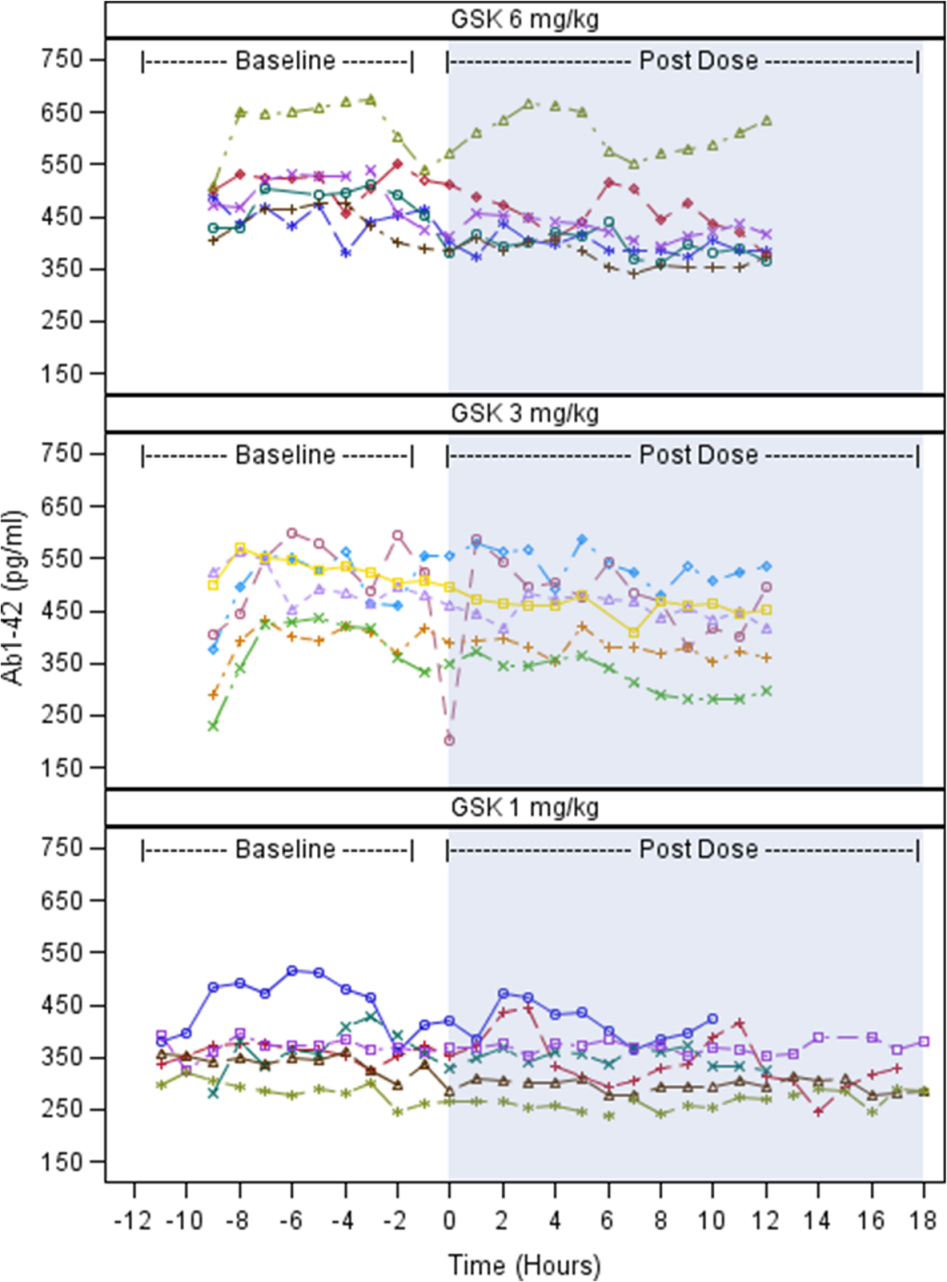
**Plots illustrating cerebrospinal fluid β-amyloid Ab(1–42) levels for individuals by time and dose level for each dose group.** GSK: GSK933776. Patients = 6 per group.

Following administration of GSK933776 1 mg/kg, 3 mg/kg and 6 mg/kg, average apparent decreases from baseline in total CSF Aβ_1–42_ (0 to 12 hours) of 22.8 pg/ml (6.2%), 43.5 pg/ml (9.2%) and 60.5 pg/ml (12.5%), respectively (Table 2). We observed greater decreases from 5 to 12 hours and lesser decreases from 0 to 4 hours compared with CSF Aβ_1–42_ (0 to 12 hours) across the dose ranges. Among individual patients, decreases from baseline in CSF Aβ_1–42_ were observed across the sampling periods after administration of GSK933776 at all dose levels. Decreases were most marked at the 6 mg/kg dose (Figure 2).

**Table 2:** **Summary statistics of decreases from baseline in cerebrospinal fluid Aβ**_**1–42 **_**following administration of GSK933776**^**a**^

**Sampling time (h)**	**GSK933776 (*n*** **= 6/dose)**	**Baseline**^**b**^	**Postdosing**	**Change (%)**	**95% CI**
0 to 4	1 mg/kg	363.4 ± 60.1	350.2 ± 61.7	−3.6	(−10.8 to 3.6)
	3 mg/kg	479.2 ± 65.8	446.4 ± 70.8	−6.8	(−14.9 to 1.2)
	6 mg/kg	502.7 ± 71.1	456.1 ± 88.9	−9.7	(−15.4 to −3.9)
5 to 12	1 mg/kg	363.4 ± 60.1	333.9 ± 52.1	−7.9	(−12.6 to −3.2)
	3 mg/kg	479.2 ± 65.8	428.9 ± 77.1	−10.7	(−20.2 to −1.2)
	6 mg/kg	502.7 ± 71.1	433.5 ± 85.1	−14.2	(−19.8 to −8.6)
0 to 12	1 mg/kg	363.4 ± 60.1	340.6 ± 55.7	−6.2	(−11.2 to −1.1)
	3 mg/kg	479.2 ± 65.8	435.7 ± 74.4	−9.2	(−17.9 to −0.5)
	6 mg/kg	502.7 ± 71.1	442.2 ± 86.4	−12.5	(−18.0 to −7.0)

^a^CI: confidence interval. Results are reported as means ± standard deviations. ^b^-6 to −1 hours predosing.

**Figure 3:**
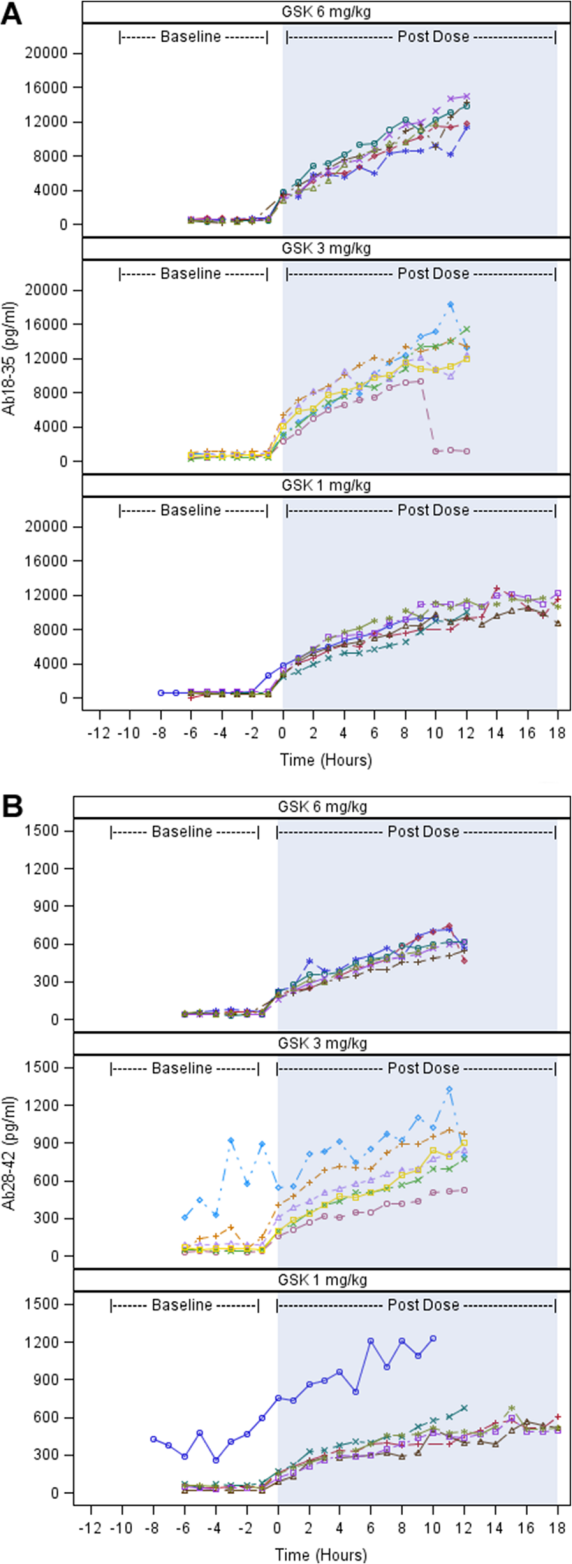
**Plots illustrating total plasma β-amyloid levels of individual patients by time and dose level. (A)** β-amyloid (18–35) Aβ_18–35_. **(B)** Aβ_28–42_. GSK: GSK933776. Patients = 6 for each dose group.

We observed increases in plasma concentrations of total Aβ_18–35_ and total Aβ42_28–42_ following administration of GSK933776 at 1 mg/kg, 3 mg/kg and 6 mg/kg doses, which began immediately after infusion (Figure 3A and 3B). Increases at the 3 mg/kg and 6 mg/kg dose levels were slightly greater than those after the 1 mg/kg dose.

Concentrations of CSF tau and phosphorylated tau 181 were variable and exhibited a small increase from baseline following administration of all three doses of GSK933776, but they did not significantly change (see Additional file 1 and Additional file 2, respectively). Three patients who received 1 mg/kg GSK933776 underwent sampling for tau for 18 hours. By 12 hours in these patients, the levels of tau appeared to have increased slightly, then plateaued and then began to decrease.

### Pharmacokinetics

Because the CSF pharmacokinetics data were minimal, median values were considered more appropriate to summarise (Table 3). The *t*_½_ parameter could not be determined, because there were insufficient CSF and plasma samples to characterise adequately the switch from the first to the terminal exponential phase. Median exposure in the CSF was higher at 6 mg/kg than at 1 mg/kg and 3 mg/kg. The value of AUC_0 to 4_ was zero at all doses because only one patient provided quantifiable values at each dose level. The pharmacokinetics profile of GSK933776 was variable in the CSF and did exhibit a dose–response relationship.

**Table 3:** **Noncompartmental pharmacokinetics in cerebrospinal fluid by dose following single dosing with GSK933776**^**a**^

**Parameters**	**Median (range)**		
	**1 mg/kg ( *N */*n*** **= 6/3)**	**3 mg/kg ( *N */*n*** **= 6/6)**	**6 mg/kg ( *N */*n*** **= 6/4)**
AUC_0–4_ (h∙mg/ml)	0.0 (0.0 to 71.1)	0.0 (0.0 to 32.1)	0.0 (0.0 to 38.9)
AUC_5–12_ (h∙mg/ml)	87.9 (12.7 to 134.9)	54.8 (6.3 to 398.8)	140.2 (49.1 to 626.5)
AUC_0–12_ (h∙mg/ml)	87.9 (12.7 to 210.9)	54.8 (6.3 to 456.0)	140.2 (49.1 to 703.9)
AUC_0–*t*_ (h∙mg/ml)	6.3 (0.0 to 339.5)	54.8 (6.3 to 456.0)	88.1 (0.0 to 703.9)
*C*_max_ (μg/ml)	12.7 (0.0 to 47.8)	20.1 (12.5 to 84.1)	28.5 (0.0 to 116.9)
*C*_t_ (μg/ml)	19.0 (11.4 to 25.3)	19.6 (12.5 to 84.1)	47.9 (18.6 to 116.1)
*t*_max_ (h)	10.0 (2.0 to 11.0)	11.8 (10.0 to 12.0)	12.0 (11.0 to 12.0)
*t*_last_ (h)	17.0 (11.0 to 19.0)	12.0 (11.6 to 12.0)	12.0 (12 to 12)
*t*_lag_ (h)	8.0 (0.0 to 10.0)	8.5 (2.0 to 11.0)	5.0 (2.0 to 8.0)

^a^AUC_0–4_: Area under the cerebrospinal fluid (CSF) concentration–time curve from time 0 to 4 hours postdosing; AUC_0–12_: Area under the CSF concentration–time curve from time 0 to 12 hours postdosing; AUC_5–12_: Area under the CSF concentration–time curve from 5 to 12 hours postdosing; AUC_0–*t*_: Area under the CSF concentration–time curve to the last quantifiable concentration; *C*_max_: Maximum CSF concentration; *C*_t_: Concentration at *t*_last_; N: Number of patients who received GSK933776; *n*: Number of patients from whom quantifiable CSF pharmacokinetics concentration levels were obtained and analysed; *t*_last_: Time of the last observed CSF concentration; *t*_max_: Time of occurrence of *C*_max_; *t*_lag_: Time prior to the first measurable (that is, nonzero) concentration in CSF.

For plasma pharmacokinetics, following intravenous infusion of GSK933776 at the 1 mg/kg, 3 mg/kg and 6 mg/kg doses, *C*_max_ values were 18.4 μg/ml, 104.0 μg/ml and 158.2 μg/ml, and *t*_max_ values were 11.7 hours, 2.1 hours and 1.9 hours, respectively (Table 4). GSK933776 exposure appeared to increase with dose level (Table 4).

**Table 4:** **Noncompartmental pharmacokinetics in plasma following single dosing with GSK933776**^**a**^

	**Geometric mean (CV in %)**					
**Parameters**	**1 mg/kg ( *N */*n*** **= 6/6)**	**95% CI**	**3 mg/kg ( *N */*n*** **= 6/6)**	**95% CI**	**6 mg/kg ( *N */*n*** **= 6/6)**	**95% CI**
AUC_0–12_ (h∙mg/ml)	0.2 (26.0)	0.1 to 0.2	1.0 (19.5)	0.9 to 1.3	1.6 (20.6)	1.3 to 2.0
AUC_0–*t*_ (h∙mg/ml)	3.3 (39.3)	2.2 to 4.9	23.0 (12.8)	20.2 to 26.3	36.3 (15.3)	31.0 to 42.6
*C*_max_ (μg/ml)	18.4 (29.0)	13.6 to 24.9	104.0 (20.0)	84.4 to 128.1	158.2 (24.0)	123.8 to 202.1
*C*_t_ (μg/ml)	0.5 (366.0)	0.1 to 2.5	1.2 (33.0)	0.9 to 1.7	1.5 (30.0)	1.1 to 2.1
*t*_last_ (h)	909.8 (105.5)	367.0 to 2,255.4	1,337.4 (0.2)	1,335.1 to 1,339.8	1,417.2 (7.0)	1,317.6 to 1,524.3
*t*_max_ (h)	11.7 (264.1)	2.6 to 53.1	2.1 (16.4)	1.8 to 2.5	1.9 (61.2)	1.1 to 3.4

^a^AUC_0–12_: Area under the plasma concentration–time curve from time 0 to 12 hours postdosing; AUC_0–*t*_: Area under the plasma concentration–time curve to the last quantifiable concentration; CI: Confidence interval; *C*_max_: Maximum plasma concentration; *C*_t_: Concentration at *t*_last_; CV: Coefficient of variation; *t*_last_: Time of last observed plasma concentration; *t*_max_: Time of occurrence of *C*_max_.

A delay in the detection of GSK933776 in CSF was observed (median values of each dose group from 5.0 hours to 8.5 hours) compared with GSK933776 in plasma, which was detected immediately. This delay was reflected in the CSF/plasma ratios, which increased with time and were in the range of 0 to 0.23%. There were no relationships between changes from baseline in CSF Aβ and any of the CSF pharmacokinetics parameters. Therefore, no PK/PD modelling was performed.

### Safety

A summary of the most frequently reported adverse events is given in Table 5. Two patients experienced serious adverse events, neither of which was considered by the investigator to be related to GSK933776. One patient who received 3 mg/kg GSK933776 experienced a procedure-related headache that started approximately 5.5 hours before administration of GSK933776. That 73-year-old patient was unable to eat and drink, was admitted to the hospital 5 days later and was given intravenous fluids and pain relief medication, and the headache resolved 8 days later. One patient required surgical treatment to remove part of a catheter that tore during placement and therefore did not receive GSK933776. That patient was admitted to the neurosurgery ward, and the disrupted piece of the catheter was removed after incision of the skin. The catheter was broken in soft tissue, and no subsequent infection or other complications occurred. The adverse event thus resolved, and the patient was discharged from the hospital the following day and was withdrawn from the study. No clinical laboratory values were reported as adverse events. One patient experienced two episodes of atrial fibrillation during GSK933776 treatment, neither of which was considered to be related to GSK933776 administration. One patient who received GSK933776 (6 mg/kg) had positive results in the binding antibody and neutralising antibody tests at the follow-up visit 56 days after the first dose.

**Table 5:** **Summary of the most frequently reported adverse events**^**a**^

	**GSK933776 dose**		
**Adverse events**	**1 mg/kg ( *N */*n*** **= 6/6)**	**3 mg/kg ( *N */*n*** **= 6/6)**	**6 mg/kg ( *N */*n*** **= 6/6)**
Any event	3 (50)	5 (83)	5 (83)
Fatigue	0	2 (33)	2 (33)
Headache	1 (17)	4 (67)	2 (33)
Nausea	0	3 (50)	1 (17)
Vomiting	0	2 (33)	1 (17)
Back pain	1 (17)	2 (33)	1 (17)
Nasopharyngitis	0	0	0
Neck pain	0	1 (17)	1 (17)
Atrial fibrillation	1 (17)	0	1 (17)
Procedural headache	0	1 (17)	1 (17)

^**a**^Data in parentheses are percentages.

## Discussion

Our findings in this study support target engagement of GSK933776 in CSF. The patients’ baseline levels of CSF Aβ_1–42_ were stable, and there were apparent decreases following all doses of GSK933776 from baseline in CSF Aβ_1–42_. These decreases seem to have been dose-dependent, with greater reductions associated with higher doses of GSK933776, and time-dependent, with greater reductions occurring 5 hours after administration. In contrast to a previous study, no diurnal variation in CSF Aβ levels was observed [14]. CSF tau levels increased slightly during this study, but the reason for this is unknown. It could be related to the total volume of CSF sampled and mobilisation of ventricular CSF to the lumbar sac. Tau concentrations in ventricular CSF are about five times higher than in lumbar CSF [15].

We observed an increase in both total Aβ_18–35_ and total Aβ42_28–42_ concentrations in plasma following GSK933776 treatment. The pharmacokinetics data confirmed the presence of GSK933776 in CSF, which suggests a central mechanistic model of action rather than a peripheral sink mechanism. Although these two theories are popular in the mechanism of action of AD vaccination, they are not mutually exclusive. Further studies are required to characterise the mechanistic model of GSK933776 mode of action early and late after treatment.

The findings regarding GSK933776 plasma pharmacokinetics in this study are consistent with those in previous solanezumab single- and multiple-dose studies [5,16]. In all of these studies, maximum drug plasma concentrations increased with drug dose levels [5,16]. Also, long terminal elimination half-lives were observed, which is typical of a monoclonal antibody with low plasma clearance. AUC_0–∞_ and *t*_½_ values were not calculated in this study, because more samples would have been required to characterise adequately the switch from the first to the terminal exponential phase. In a previous study of GSK9933776, however, a long terminal elimination half-life (average range: 10 to 15 days) was observed (manuscript in submission). The increase in plasma levels of total Aβ_18–35_ and Aβ_28–42_ did not exhibit an apparent dose–response relationship during the short observation time in this study.

This study confirms the presence of GSK933776 in CSF. CSF versus plasma ratios increased with time and were ranged from 0 to 0.23%, reflecting the delay of detection of GSK933776 in CSF by 5.0 hours to 8.5 hours (median values in dose groups).

This study has several limitations. The decreases from baseline in CSF Aβ_1–42_ were greater with the higher dose of GSK933776 (6 mg/kg) than with the 1 mg/kg and 3 mg/kg dose groups. However, this result might have been driven by the higher baseline CSF Aβ_1–42_ values in the 6 mg/kg group compared with the other groups. Differing baseline CSF Aβ_1–42_ values were also observed between the other dose groups. However, all dose groups showed similar trends in CSF Aβ_1–42_ changes over time, regardless of baseline CSF Aβ_1–42_ values, suggesting that this limitation did not affect the interpretability of the data. This study also has a small sample size and is not formally powered; therefore, we conducted no formal statistical analyses. Despite the feasibility-driven limited sample size in this study, our sample size is similar to those of other published studies in the field [6,7]. In addition, it is unknown to what extent GSK933776 may have interfered with Aβ in the CSF Aβ_1–42_ assay. The potential for spurious results induced by any possible interaction of GSK933776 with the CSF Aβ_1–42_ assay was not assessed, thus limiting the interpretation of the results. However, there was no apparent relationship between changes from baseline in CSF Aβ and any CSF pharmacokinetics parameters. The differences in mean baseline CSF Aβ levels observed between the dose groups were most likely due to chance resulting from the small sample size.

The observed frequency of *APOE* ϵ4 carriers was higher in this study (14 (93%) of 15 patients) than in a typical AD population (64.8%) [17]. This difference could be due to the small number of patients in this study and the screening selection criteria pertaining to reductions in Aβ and increases in tau levels in the CSF. Other contributing factors could be the age of the patients and/or the MMSE score ranges. In this study, no *APOE* ϵ4 data were obtained from patients who failed screening, because pharmacogenetic samples were taken only from patients who were randomised.

We believe that, to the best of our knowledge, this study is one of very few intervention studies in which the feasibility of using the CSF catheter to assess short-term Aβ changes has been investigated. One catheter-related serious adverse event occurred during the catheter insertion procedure in our study.

The outcomes of phase III studies conducted with the Aβ monoclonal antibodies bapineuzumab and gantenerumab, as well as another pivotal study carried out with solanezumab, have failed to demonstrate unambiguous efficacy in patients with mild to moderate AD, but they have triggered significant new activity in the study of earlier stages of AD.

## Conclusions

The results of this study support pharmacological activity and target engagement of GSK933776 in both CSF and plasma. They demonstrate that the continuous sampling method using a catheter can be used successfully to assess Aβ changes after single-dose administration of an anti-Aβ antibody in patients with mild AD or MCI.

## Abbreviations

AD: Alzheimer’s disease; Aβ: β-amyloid; AUC0–12: Area under the plasma and cerebral spinal fluid concentration–time curve from time 0 to 12 hours postdosing; AUC0–4: Area under the cerebral spinal fluid concentration–time curve from time 0 to 4 hours postdosing; AUC0–t: Area under the plasma and cerebral spinal fluid concentration–time curve to the last quantifiable concentration; AUC5–12: Area under the plasma and cerebral spinal fluid concentration time curve from 5 hours to 12 hours postdosing; Cmax: First occurrence of the maximum observed plasma and cerebral spinal fluid concentration determined directly from raw concentration time data; CNS: Central nervous system; CSF: Cerebrospinal fluid; Ct: Concentration at t_last_; ECG: Electrocardiogram; MCI: Mild cognitive impairment; MMSE: Mini Mental State Examination; MRI: Magnetic resonance imaging; tlag: Time prior to the first measurable (nonzero) concentration in cerebral spinal fluid; tlast: Time of the last observed plasma concentration; tmax: Time at which C_max_ was observed determined directly from raw concentration time data.

## Competing interests

MS, XT, AY, SK, AL, MC, CH, JH and PM are GlaxoSmithKline employees. This study was funded by GlaxoSmithKline. TL, NA, AR, CAFvA and HZ were sponsored by GlaxoSmithKline. GlaxoSmithKline sponsored and paid for this study.

## Authors’ contributions

TL participated in the recruitment of study participants, partly performed the study and reviewed the data. AL participated in the acquisition, analysis and interpretation of pharmacodynamic data. HZ contributed to the study concept and design, biochemical analyses and critical review of the manuscript for important intellectual content. AY participated in the delivery and interpretation of genetic data (*APOE* ϵ4). XT contributed to the study concept and design, partly performed the study and acquired the data. NA participated in the study design, performed the study and obtained and interpreted the data. CAFvA participated in the recruitment of study participants, partly performed the study and reviewed the data. SK was the project manager and reviewed patient data. AR generated and reviewed patient data. MC reviewed safety data. PM participated in the design of the study, performed statistical analyses and provided interpretation of the data. CH contributed to and reviewed the pharmacokinetics bioanalytical data. MS performed mathematical analysis and reviewed pharmacokinetic data. JH contributed to the design and conduct of the study. All authors read and approved the final manuscript.

## Supplementary Material

Additional file 1 **Individual patient plots of cerebrospinal fluid tau by time and dose level.** GSK: GSK933776. Coloured dashed lines refer to each individual patient plot. Click here for file

Additional file 2 **Individual patient plots of cerebrospinal fluid phosphorylated tau 181 by time and dose level.** GSK: GSK933776; P-TAU: Phosphorylated tau. Coloured dashed line refers to each individual patient plot. Click here for file
