# Supplementary figures and images for: Modulation of β-amyloid by a single dose of GSK933776 in patients with mild Alzheimer’s disease: a phase I study
Source: Alzheimers Res Ther. 2014 Apr 9;6(2):19. doi: 10.1186/alzrt249 (PMC4055052; doi:10.1186/alzrt249)

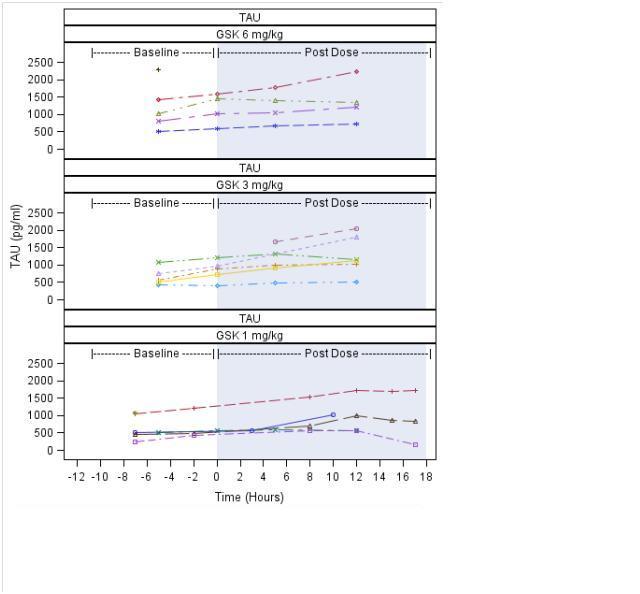

Supplement: Additional file 1 — Individual patient plots of cerebrospinal fluid tau by time and dose level. GSK: GSK933776. Coloured dashed lines refer to each individual patient plot. [file alzrt249-S1.jpeg]

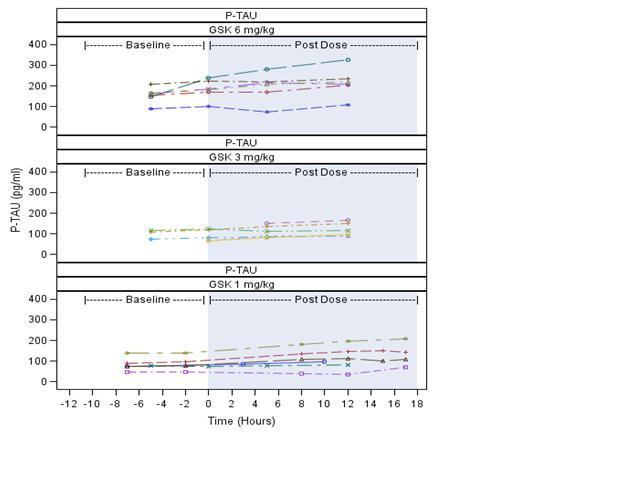

Supplement: Additional file 2 — Individual patient plots of cerebrospinal fluid phosphorylated tau 181 by time and dose level. GSK: GSK933776; P-TAU: Phosphorylated tau. Coloured dashed line refers to each individual patient plot. [file alzrt249-S2.jpeg]
